# Microbial Contamination and Antibiotic Resistance in Marketed Food in Bangladesh: Current Situation and Possible Improvements

**DOI:** 10.3390/antibiotics12030555

**Published:** 2023-03-10

**Authors:** Mohammed Abdus Samad, Linnea Eberson, Ruhena Begum, Mohammad Gazi Shah Alam, Faisol Talukdar, Rahima Akter, Sinh Dang-Xuan, Garima Sharma, Shariful Islam, Nure Alam Siddiky, ASM Ashab Uddin, Mohammad Asheak Mahmud, Md Samun Sarker, Md. Siddiqur Rahman, Delia Grace, Johanna F. Lindahl

**Affiliations:** 1 Antimicrobial Resistance Action Center, Bangladesh Livestock Research Institute, Savar, Dhaka 1341, Bangladesh; 2 Department of Clinical Sciences, Swedish University of Agricultural Sciences, 75007 Uppsala, Sweden; 3 Department of Livestock Services, Krishi Khamar Sarak, Dhaka 1215, Bangladesh; 4 Department of Biosciences, International Livestock Research Institute, Hanoi 100000, Vietnam; 5 Department of Biosciences, International Livestock Research Institute, Nairobi 00100, Kenya; 6 Department of Medical Biochemistry and Microbiology, Uppsala University, 75123 Uppsala, Sweden; 7 Department of Medicine, Faculty of Veterinary Science, Bangladesh Agricultural University, Mymensingh 2202, Bangladesh; 8 Food and Markets Department, Natural Resources Institute, University of Greenwich, Maritime, Chatham ME4 4TB, UK

**Keywords:** antimicrobial resistance, South Asia, antimicrobial use, food safety, multidrug-resistance (MDR), low-and middle-income countries

## Abstract

Antimicrobial resistance (AMR) is a public health problem worldwide. Bangladesh, like its neighboring countries, faces many public health challenges, including access to safe food, inadequate food surveillance, as well as increasing AMR. This study investigated bacterial contamination and the AMR profile of pathogens in marketed food in Bangladesh and explored barriers to reducing AMR in the country. We collected 366 tomatoes, 359 chicken and 249 fish samples from 732 vendors in traditional markets in urban, peri-urban and rural areas in Bangladesh, as well as from 121 modern retails in Dhaka capital to analyse *Vibrio cholerae* and *Escherichia coli* in fish, *Salmonella* in chicken, and *Salmonella* and *E. coli* in tomatoes. Antibiotic susceptibility against 11 antibiotics was tested using a disc diffusion test and interpreted by an automated zone inhibition reader. In addition, a qualitative study using key informant interviews was conducted to explore antimicrobial use and AMR reduction potential in Bangladesh. We found *E. coli* in 14.21% of tomatoes and 26.91% of fish samples, while 7.38% of tomatoes and 17.27% of chicken were positive for *Salmonella*, and 44.98% of fish were positive for *Vibrio cholerae*. In total 231/319 (72.4%) of all pathogens isolated were multidrug-resistant (MDR) (resistant to three or more antibiotic groups). Qualitative interviews revealed an inadequate surveillance system for antibiotic use and AMR in Bangladesh, especially in the agriculture sector. To be able to fully understand the human health risks from bacterial hazards in the food and the AMR situation in Bangladesh, a nationwide study with a one health approach should be conducted, within all sectors, including AMR testing as well as assessment of the antimicrobial use and its drivers.

## 1. Introduction

Antimicrobial resistance (AMR) is considered one of the most important threats to public health globally [1,2]. Antibiotics have been excessively used, not only within human health care, but also within agriculture, veterinary medicine, and as animal growth promoters, contributing to health advances while also improving animal welfare [3]. The extent of antibiotic use varies between countries due to regulations in place, policies, knowledge, and income levels [4,5], and many of the antibiotics critical for human health care are used within the livestock sector [3,6]. Low- and middle-income countries (LMICs) face challenges like inadequate health care systems, high accessibility to over-the-counter drugs (OTCs), low biosecurity, and unsafe food systems, which all contribute to high and unregulated use of antibiotics [5,7,8]. For example, 60% of poultry farmers use antibiotics without prescriptions in Bangladesh [9,10]. Additionally, a lack of awareness about antimicrobials and poorly developed surveillance programmes, especially within the veterinary sector [11,12], makes it hard to fully understand the situation both regarding antimicrobial use (AMU) and AMR [2,5].

In Asia, AMR is growing faster than in any other region in the world [10]. Bangladesh is one of the most densely populated countries in the world and shares many problems common to LMICs [13]. People tend to live close to their animals, creating an environment where zoonotic diseases and AMR can spill over [14,15]. Bangladesh, like many of its neighboring countries in Asia, faces the problem of unregulated AMU [13]. Food safety is also a major concern to public health in the country, and has been inadequately addressed [16,17]. Within the country, there is little information about pathogens in foodstuff and no national surveillance of AMU and AMR [12]. As a result, there is a paucity of evidence about current food safety hazards and the AMR-pattern of contaminating pathogens. Furthermore, it raises the question of how well people are aware of the problems with AMU and AMR in Bangladesh, and the solutions for this situation [13]. The Bangladesh National Action Plan (BNAP) for containment of AMR, endorsed in 2017, covers the country’s policies for improving AMU and AMR through multisectoral measures such as implementing rational AMU, surveillance of AMR, and enforcing present laws [18], and there is a new National Antimicrobial Resistance (AMR) Strategy of Bangladesh which intends to build a robust, cross-sectoral AMR surveillance system to influence policy and antimicrobial production, distribution, and usage [19]. The BNAP is aligned with the Global Action Plan (GAP) issued by WHO in 2015 and is complemented by a roadmap [18]. A study by Orubu et al. [20] has evaluated the BNAP by comparing it to the GAP and found that BNAP aligns well with the GAP, but some policy gaps regarding financing modality, specifications for AMR stewardship in the veterinary sector, and frameworks for monitoring and evaluation were found. They concluded that these gaps must be addressed for successful veterinary AMR containment. In Bangladesh, due to an inadequate animal healthcare system, informal healthcare providers are frequently being contacted when animals get sick [21]. Poor infrastructure is cited as one reason for villagers not receiving appropriate help from a government veterinarian, but it also appears that villagers would rather contact a drug seller or pharmacy than a government veterinarian to get medicine for their animals since the veterinarian would charge them more [22]. Antibiotic resistance is exacerbated in LMICs by the widespread availability of over-the-counter drugs without a prescription [5]. This commonly occurs also in Bangladesh, and studies have shown that both prescription of antibiotics and use without a prescription are inappropriate in many cases [13,23]. Further complicating the situation in Bangladesh is a large number of drug sellers and unlicensed drug sellers [23,24]. Practically all retailers distribute antibiotics, but only a few have educated staff [23,25].

Bacteria, including resistant bacteria, can be transmitted from animals to humans directly, or via contamination of food and water. While livestock products are common sources of zoonotic bacteria, vegetable contamination with different types of multidrug-resistant (MDR) bacteria is also common in Bangladesh [26]. Similarly, *Vibrio cholerae* contamination is a problem in fish [27], and this bacterium continues to cause severe disease with frequent outbreaks in Bangladesh [28,29]. This study aimed at assessing the prevalence of selected foodborne hazards and the presence of AMR in chicken, fish, and tomatoes marketed in Bangladesh, as well as identifying common barriers to reducing AMR in the country.

## 2. Results

### 2.1. General Characteristics of Food Vendors and Shops

Traditional markets were identified at all study sites, while supermarkets, called “supershops”, were found only in urban areas. The total number of urban samples was 467 (242 samples from supershops and 225 from traditional markets). The number of samples from the peri-urban area was 255 and from rural 252. The 974 samples were collected from food sold in stalls of 853 different vendors. The vendors, i.e., respondents, were interviewed using a pre-tested questionnaire, and the enumerator also made notes on which products were sold in the stall and how the products were stored (Table 1).

Most of the interviewed vendors were men (98.2%, 838/853), and only 1.8% were women. About half of the vendors (50.3%) were in the age group 20–35, and there was no statistically significant difference in age between males and females. Thirty-eight percent of vendors completed primary school, followed by 25.6 % that had completed grade 5–10. The highest education completed differed between men and women, where 33.3% of the women had tertiary education, in contrast to 7.3% of men. Among vendors working in supershops, almost all women reported “graduation and above” as the highest education completed. Among women working in traditional markets, 7/9 had no education, which was a significantly higher proportion than men (*p* = 0.034).

Most of the samples and participants came from traditional markets. However, among women, there were only a few more participants from traditional markets (60.0%) than from supershops (40.0%), while among men the vast majority were from traditional markets (traditional 86.3% and supershop 13.7%). Overall, it was significantly more common that a supershop worker was female than a worker in a traditional market (*p* = 0.004). The distribution of men and women at different study sites showed no significant difference in statistical analysis.

There was a significant difference (*p* < 0.001) between products sold by women and men. Only men sold fish, and the rest of the men sold tomatoes (37.0%) and chicken (37.6%). Most women, 86.7%, sold tomatoes, and 13.3% sold chicken (Table 1). The odds of the vendor being a female were 6.6 times higher (95% CI 1.5–29.5, *p* < 0.013) for tomatoes than for chicken.

Of the products sold, 711 (83.4%) were not on ice in the stall, while 142 (16.7%) were cooled to some extent (Table 1). Cooling practices did not vary significantly by gender, however, there was a significant difference (*p* < 0.001) between products. Fish was more likely to be cooled as compared to chicken (OR 3.6, 95% CI 2.39–5.53, *p* < 0.001), and tomatoes were less likely to be cooled than chicken (OR 0.3, 95% CI 0.19–0.60, *p* < 0.001). Most influential was the type of shop, where supershops were more likely to have cold products compared to traditional markets (OR 31.5, 95% CI 19.4–51.0, *p* < 0.001). Data showed that in supershops, 73/74 fish were held at low temperatures. Compared to rural markets, markets in peri-urban areas were less likely to have cold products (OR 0.1, 95% CI 0.05–0.32, *p* < 0.001) and shops in urban areas were more likely to have cold products (OR 2.6, 95% CI 1.69–3.97, *p* <0.001).

### 2.2. Microbial Contamination in Food

Of the 974 samples taken, 615 were analysed for two different bacteria (fish analyzed for *Escherichia coli* and *V. cholerae*, and tomatoes analyzed for *Salmonella* spp. and *E. coli*) and 359 for one bacterium (chicken only analyzed for *Salmonella* spp.). Of the total number of cultivations, 320 (20.1%) were positive (Table 2), with fish being the most frequently positive food product.

In total, 89 samples were positive for *Salmonella* spp. (Table 2), representing 12.3% of total analyses done for the bacterium, while for *E. coli*, 119 (19.4%) were positive, and for *V. cholerae* 112 (45.0%). All areas had positive samples, but the peri-urban area had a higher proportion of tomatoes positive for *E. coli* and rural areas had a higher proportion of chicken positive for *Salmonella* (Table 2).

Samples were less likely to be positive for *Salmonella* spp. if they came from the peri-urban or urban area (OR 0.3, 95% CI 0.18–0.63, *p* = 0.001 resp. OR 0.4, 95% CI 0.24–0.65, *p* < 0.001) compared to the rural area, and if the product was tomato compared to chicken (OR =0.4, 95% CI 0.23–0.61, *p* < 0.001). For *E. coli*, the odds ratio for a positive cultivation was 3.6 times higher (95% CI 1.56–8.08, *p* = 0.003) than if the sample came from a traditional market. If the sample came from a peri-urban area, it was also more likely to be positive for *E. coli* (OR 2.4, 95% CI 1.37–4.31, *p* = 0.002), and if the sample was from fish, the odds ratio for being positive was 2.5 times higher (OR 2.5, 95% CI 1.64–3.83, *p* < 0.001). There was no significant difference in the proportion of samples positive for *Salmonella* spp. or *E. coli* depending on if the product was kept cool.

### 2.3. Quantification of Bacteria

Total coliform count (TCC) analyses were performed on a subgroup of fish and tomato samples and the colony-forming units (CFU) in the samples varied from 0 to too numerous to count (TNTC). For analytical purposes, the logarithmic values (LogCFU) were calculated. The values classified as 0 or TNTC were set as missing values in order to use the variable in statistical analysis. LogCFU/g of TCC were significantly higher in traditional markets compared to supershops (4.44 and 3.98, respectively, *p* = 0.0001) and the LogCFU/g of TCC in tomatoes were significantly higher than in fish (4.47 and 4.17, respectively, *p* = 0.002). Even though not significantly different, the LogCFU/g was slightly higher in products that were not cooled compared to products that were cooled (4.40 and 4.21 LogCFU/g, respectively, *p* = 0.087).

### 2.4. Antimicrobial Resistance

A total of 319 samples were tested for antimicrobial susceptibility through standard disc diffusion tests. Susceptibility for amoxicillin + clavulanic acid (AMC), cefixime (CFM), ceftriaxone (CRO), chloramphenicol (C), erythromycin (E), gentamicin (CN), streptomycin (S), penicillin G (*p*), tetracycline (TET), sulfamethoxazole + trimethoprim (SXT) and nalidixic acid (NA) was tested in the panel. CFM and CRO, as well as CN and S, were counted as having the same antimicrobial spectra, i.e., classed as the same group. All the other antibiotics were counted as separate groups. The 319 samples were categorized as sensitive (S), intermediate (I), and resistant (R) to each of the tested antibiotics in the panel, based on disc diffusion cut-off values for each antibiotic (Table 3).

Among *Salmonella* isolates from tomatoes, resistance was highest for penicillin G (74.1%), followed by erythromycin (63.0%) and tetracycline (63.0%). *E. coli* isolates from tomatoes had a similar pattern: 100% resistance to penicillin G and 68.6% to erythromycin and tetracycline. However, the second highest percentage of resistance was found against streptomycin (78.4%). *Salmonella* isolates from chicken showed the highest values of resistance for penicillin G (96.8%), followed by tetracycline (93.6%) and erythromycin (87.1%), but were also high for sulfamethoxazole+ trimethoprim (71.0%). Regarding samples from fish, the highest percentage of resistance was to penicillin G (100%) for both *Vibrio* and *E. coli*, followed by 65.7% resistance to streptomycin and 68.7% to tetracycline for *E. coli*, and to streptomycin and erythromycin (34.8% resp. 32.1%) for *V. cholerae*. *Vibrio* isolates from fish had only one category, penicillin, where the proportion of resistance exceeded 50% (Table 4).

Bacterial isolates that were resistant to three or more antibiotic groups were classified as being MDR. The number of isolates classified in this group is displayed in Table 5. *Salmonella* spp. and *E. coli* are considered to be naturally resistant to penicillin G, and resistance to this antibiotic is therefore not included in the MDR classification.

Of the 89 samples positive for *Salmonella* spp., 75 of those tested for antimicrobial susceptibility were classified as MDR, representing 84.3% of all positive samples for the bacterium. Corresponding numbers for *E. coli* and *V. cholerae* were 106 out of 118 (89.8%) and 50 out of 112 (44.6%) respectively.

In total, 72.4% of all bacteria strains were classified as being MDR, with most MDR among samples from chicken isolates positive for *Salmonella* spp (95.2% of positive cultivations classified as MDR) followed by tomato analysed for *E. coli*, where 94.1% of positive cultivations classified as MDR. For fish samples analysed for both *V. cholerae* and *E. coli*, the percentage of MDR isolates was 44.6% and 86.6%, respectively.

For *V. cholerae*, there was a significant difference (*p* < 0.001) in MDR between samples from different study sites, with the isolates from the urban area having 25.8% MDR, while in the peri-urban the MDR proportion was 88.5%, and in the rural area it was 45.8%. There was also a significantly lower percentage of MDR among the isolates of *V. cholerae* from supershops (25.7%) compared to traditional markets (53.2%, *p* = 0.007). No significant differences between study sites were found among samples positive for *Salmonella* and *E. coli*. For *Salmonella*-positive samples, there was a significant difference (*p* < 0.001) in the proportion of MDR, which was higher in chicken than in tomato (Table 5).

A multiple-antibiotic resistance (MAR) index was calculated as the proportion of the tested antibiotics that the bacteria were resistant against (Table 6). The MAR index is found by dividing the number of antibiotics to which an isolate is resistant by the total number of antibiotics to which the organism is exposed. A MAR of more than 0.2 indicates high contamination [7,30]. The proportion of isolates with a MAR index greater than 0.25 was 74.0% (236/319). Five isolates had a MAR index above 0.75.

### 2.5. Qualitative Results on AMU Situation and AMR Prevention in Bangladesh

**Figure 1:**
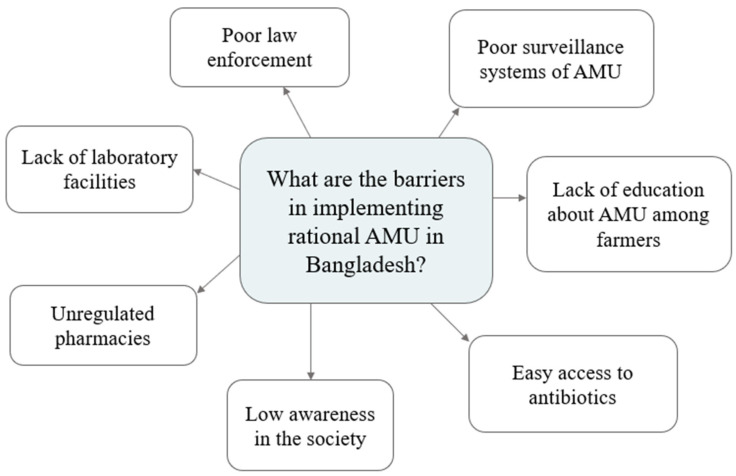
Identifying barriers to implementing rational AMU in Bangladesh by respondents in key informant interviews.

All key informants (KI) classified AMR as a national problem in Bangladesh. Many of the participating persons mentioned “lack of education”, “not enough enforcement of laws”, “not enough cooperation between different actors” and “unregulated pharmacies” (Figure 1) as major problems both regarding AMU and AMR. Other aspects such as “not enough laboratory facilities” and “lack of knowledge on how to use laboratory facilities” were also considered contributing factors to both the AMU and limited AMR surveillance in Bangladesh.

Everyone putting AMR as a nationwide problem thought that the situation could be improved, and all respondents also agreed that better and more adequate surveillance programmes needed to be developed. To this end, respondents noted “the infrastructure and logistics can be hard” and “the veterinary coverage is not good enough”. Other stakeholders suggested cooperation and working with a one health perspective as solutions to putting together nationwide surveillance, preferably for both human and animal health. When asked questions about current guidelines for AMU, the answers varied from “do not know if there is for both humans and animals, but for humans, we are developing some now” to “I think there is, but no one can follow that”.

**Figure 2:**
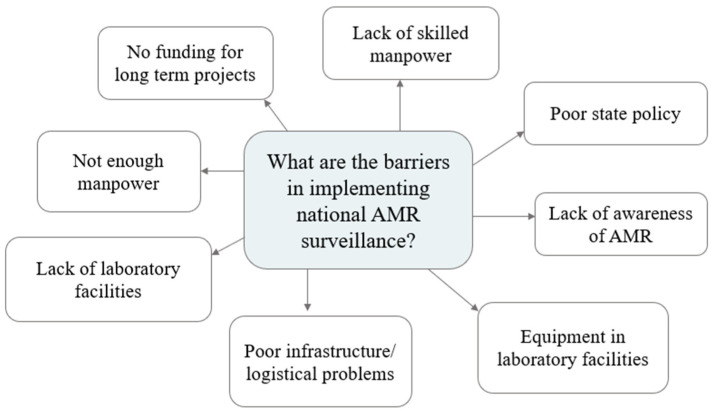
Various barriers in implementing national AMR surveillance.

Among the people interviewed, most considered the government was responsible for enforcing laws and drafting new guidelines or formulating surveillance programmes. Many stakeholders added that private companies also had a responsibility to set an example. Another interviewee said that “professors interested in creating publications care about this, but what difference does it make”, meaning that many studies are being made, without changing practices. Others said that regarding facilities for testing for AMR, there was a lack of management within the public sector, leading to facilities not operating. The various barriers to achieving national AMR surveillance are shown in Figure 2.

Just one of the interviewees repeatedly stressed preventive work at farms as a solution for decreasing the AMU. This interviewee believed that antibiotics are necessary and should not be ruled out, but that preventive work should be the major strategy. Another interviewee pointed to more testing, and above all, always testing before a change of antibiotic in case of failure of treatment as important.

Overall, the common picture among KII was that AMU and AMR are problems in Bangladesh, and that action against these problems needs to be taken. Many respondents spoke about current laws in place for regulating AMU, which state that no antibiotics can be bought without a prescription and that antimicrobial growth promoters are regulated. They also mentioned laws regulating maximum residue levels (MRL) of antibiotics in food products. Other things pointed out were drugs of poor quality and incorrect dosage and treatment lengths when antibiotics were used. All of these statements and facts leading back to two of the most frequent opinions “raise awareness” and “better law enforcement”. According to one of the respondents, the goal should be “no prescription, no antibiotics” within all sectors.

## 3. Discussion

This cross-sectional analysis of food products at markets in three different areas showed frequent contamination with pathogens and the occurrence of MDR in marketed food. The food products chosen are among the most commonly consumed in Bangladesh but the contamination levels of food safety hazards have been little studied, and they are produced in environments where antibiotics are potentially overused, resulting in risks of exposing consumers to AMR. Both products intended for cooking and those intended for eating raw were included; the former could result in a lower bacterial burden for the consumer. Nonetheless, handling the product itself may be a source of microbial contact, and cross-contamination is a possibility [31].

Within this study, the prevalence of three different bacteria (*Salmonella, E. coli,* and *V. cholerae*) and their antimicrobial susceptibility were investigated. From the 974 samples, 1589 cultivations were made, of which 320 were positive for bacteria. The majority of bacterial isolates were classified as MDR (231/319, 72.41%). Since food products and environmental aspects have been proposed as sources for transmitting resistance genes [32,33,34], findings of MDR bacterial strains in animal food products are a concern and further strengthen these theories [33,35,36]. The lowest proportion of MDR bacteria was found in *V. cholerae* from fish, where almost 45% of isolates were MDR. For *E. coli* isolates from tomato and *Salmonella* isolates from chicken, the proportion MDR exceeded 90%. The proportion of isolates with a MAR index greater than 0.25 was almost 74%. These results show that a higher percentage of isolates are probably from high-risk sources and come from settings where several antibiotics are used.

The panel of antibiotics included in sensitivity testing was selected by the Bangladesh Livestock Research Institute (BLRI), based on standard procedures. Out of the eleven antibiotics used in screening for resistance in this study, four different antibiotic substances were classified within two groups (cefixime and ceftriaxone as cephalosporins within the beta-lactams, and gentamicin and streptomycin within the aminoglycosides), and this study considered resistance to both antibiotics in one group as the same when calculating MDR.

The study covered not only animal-derived foods, but also tomato samples. Most bacterial isolates from tomatoes were classified as MDR. Without knowing the exact cause for this, factors such as contamination from the environment, other products, or due to poor hygiene among vendors could contribute to the contamination of tomatoes [37]. Environment and the food handlers could also be the source of positive samples among chicken and fish, and could possibly vary between different study sites since movement and a number of people, facilities for keeping good hygiene, and movement of live animals, etc. In comparison to poultry and fish, tomatoes are more likely to be consumed raw, and may therefore pose a higher risk to consumers.

Logarithmic values for colony-forming units (LogCFU) of total coliform count (TCC) were calculated for a subgroup of samples. There was a wide range of values, from zero to too numerous to count. Selected food samples are unlikely to have a coliform count of zero, since coliform bacteria is one of the most common indicator bacteria, often present in food [38,39], and thus a value of zero could be suspected to be due to an error, and those results were set as missing values in the analyses. Values that were too numerous to count were also removed from the statistical analysis, which might induce bias in the result, however, this was only the case for three samples. There was a significantly higher TCC at traditional markets than at supershops in our study. This could maybe be explained by the high correlation between cooling and supershops, or by better hygiene. It is well-known that cooling of products leads to less bacterial growth, and while it might be expected that modern retail has lower levels of contamination, but this has not always been the case [40]. Approximately 83% of the samples were not cooled, and fish were more likely to be kept cold than chicken and tomato, reflecting the ease of spoilage. It was also seen that traditional markets were rarely cooling off products compared to modern urban outlets. Surprisingly, peri-urban areas had less cooling than rural areas, which could explain the relatively high prevalence of bacteria within peri-urban food samples. TCC was also significantly higher in tomato than in fish, which might be explained by the fact that tomato was not kept as cool as fish. It is worth mentioning that, besides cooling of products, the degree of original contamination of the product will also affect the contamination levels found later. The overall results of bacterial contamination were aligned with other studies conducted in other study areas discussed in an article by Ahmed et al. [15], and it also correlates with a high proportion of reported AMR in LMICs [9,41].

A statistical difference was found among the distribution of vendors, where more women sold tomatoes, and only men sold fish, and this kind of difference could be due to socioeconomic standards, traditions, or other variables within the country. However, no significant difference in the prevalence of bacteria was related to gender in our study. The influence of gender on food safety is little understood. A study from Nigeria found that food sold by women had a lower bacterial contamination [42].

Findings from key informant interviews regarding antimicrobial use were similar to studies carried out in other LMICs, highlighting the high proportion of OTC sales of antibiotics, poor surveillance, and lack of awareness [12,41]. Other studies [13,21] have shown inappropriate sales of antibiotics in Bangladesh, and this was identified as a barrier to reducing AMR. These factors contribute to antimicrobial use and AMR within the country [15]. Numerous studies have been conducted in the area of OTC antibiotic sales in an effort to understand why this route of administration is so common. A low understanding of AMR, pharmacist education, and the fact that pharmacies are frequently the first point of contact were all cited as potential explanations. [23,24]. At least two of these three factors were also mentioned in KIIs as contributing to high OTC sales.

Antimicrobial resistance is a rapidly developing issue that is extremely concerning for public health globally [1,43]. MDR bacteria are reported from multiple countries [41], and the increasing movement of people, animals, and food products around the world complicates the scenario [44]. Reports of resistance genes spreading worldwide, indicate a slow, neglected pandemic [45]. Resistance in some enteric bacteria has been well documented over a long time, and studies indicate low resistance even before antimicrobials were introduced [46], while more recent reports indicate higher resistance levels [12]. Overall, the problem requires solutions, which need to include both more prudent use of existing antibiotics, as well as the development of new ones and alternatives [46,47].

Currently, there are laws and regulations about AMU in Bangladesh, which also were mentioned by key informants, however, they are not being adhered to [13,20,48]. Bangladesh banned the use of antibiotics in animal feed production by enacting “The Fish Feed and Animal Feed Act, 2010” [49] and “The Animal Feed Rule, 2013” [50]. Recently the Directorate General of Drug Administration (DGDA) banned colistin use in animal health at its 253rd meeting [51]. The key informants attributed this to disregard, lack of knowledge, or logistical difficulties. Poor infrastructure and inadequate health care are regarded as contributing factors in this situation in other LMICs as well [52,53]. Within the country, there is also a lack of national ongoing surveillance programmes for both AMR and AMU, especially within the veterinary sector [12,20]. Guidelines related to AMR surveillance were under development for human health care according to one key informant, but for the animal sector, none of the key informants could recall anything about guidelines. This indicates that present guidelines might be unknown to the population. Prudent antimicrobial use and smaller studies depicting the AMR situation are some of the aspects considered important by the WHO for improving the AMR problem [54]. However, lack of funding for projects and availability of manpower and facilities may hinder the implementation according to the key informants.

In addition to implementing judicious antibiotic use across all sectors, another factor to take into account is antibiotic production. Large-scale production of antibiotics takes place in countries neighboring Bangladesh [55]. Waste from this production, i.e., antibiotic residues, could also be a source of resistance that complicates the situation in Bangladesh. This kind of aspect points out that the resistance problem knows no borders and that countries might have a situation that cannot be controlled or improved just by implementing restrictions within the country.

Bacterial contamination in the food products sampled in this study could be from a variety of sources. Possible origins could be contamination from surroundings by other foodstuffs, the vendor, surfaces, or contamination at slaughter. It is impossible to say where bacteria possibly carrying AMR were introduced to the sampled material. To further evaluate the origin, research through the entire value chain would be required. These could also be complemented by studies of bacterial flora in humans that have consumed previously sampled products. Bacterial cultivation could also be complemented by molecular methods. Things such as the storage of samples before analysis and transport can also affect the result, and even if the goal was to transport all samples in a cooling box in our study, storage time could affect the results, particularly the bacterial count.

The situation in Bangladesh regarding AMR is not fully understood given the absence of comprehensive, national, long-term surveillance. AMU and its unregulated nature within the country, both within human, veterinary, and agricultural sectors, contribute to the growth of AMR. Even though it is not possible to say what is the most significant contributing part to the situation in Bangladesh, it is likely that all these different components interact with each other and create the situation. Furthermore, neighboring countries with antibiotic production might contribute to the overall AMR picture through poor waste management, making the situation more complex.

To better understand the whole picture in Bangladesh, national AMR surveillance is slowly starting. To slow the spread of AMR, rational AMU would have to be implemented urgently. A greater public understanding of antibiotics and AMR could help to change the situation. In addition, because the problem has no borders, more global action is required to establish a more prudent use of antibiotics.

## 4. Materials and Methods

Ethical approval was given by International Livestock Research Institute (ILRI) Institutional Research Ethics Committee (IREC) with approval number 2018-27. All participants were given information and gave written informed consent.

### 4.1. Study Design and Areas

#### 4.1.1. Study Design

This was a cross-sectional study of microbial food contamination, AMR-pattern mapping, and vendor interviews based on previously conducted fieldwork in Bangladesh from November 2018 to June 2019. The fieldwork, including laboratory work, was done by BLRI, Bangladesh Food Safety Authority (BFSA), International Livestock Research Institute (ILRI), and International Food Policy Research Institute (IFPRI). Complementary interviews, and descriptive and statistical analysis of data from previously collected material mentioned above were done during November and December 2020.

#### 4.1.2. Study Areas

**Figure 3:**
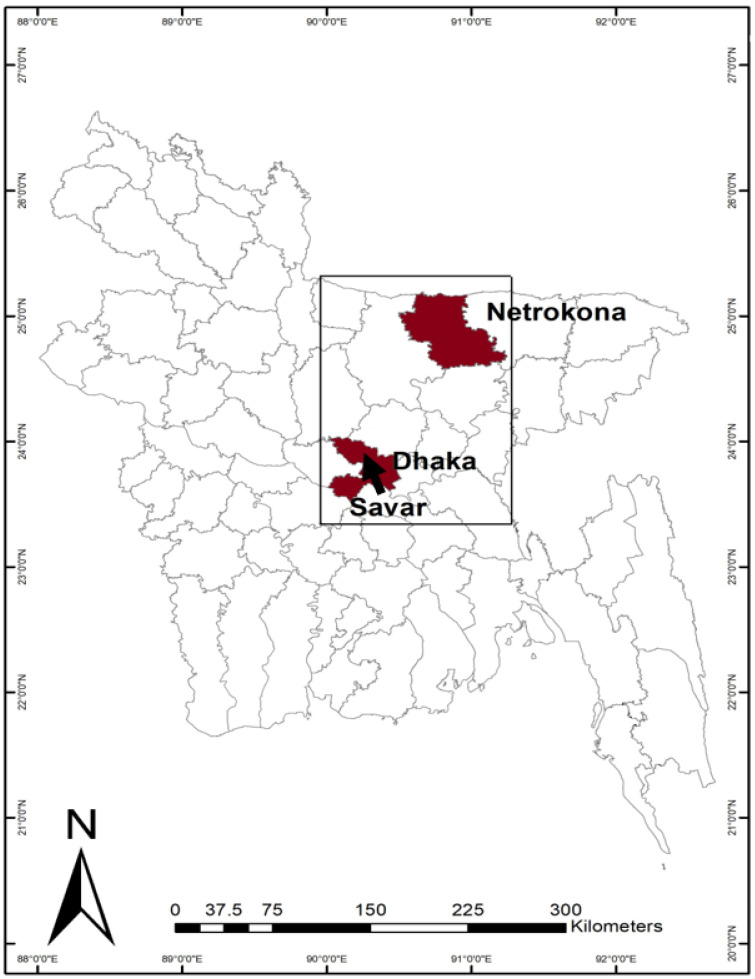
Map of Bangladesh including sites for sampling, showing the Netrokona and Dhaka district. The location of Savar within the Dhaka district is indicated by an arrow.

A total number of 974 samples were collected from 853 different vendors in Dhaka city (urban), Savar (peri-urban), and Netrokona (rural) district, displayed in Figure 3. In Dhaka, samples came from both traditional markets and supermarkets, and in Savar and Netrokona districts samples came from traditional markets.

### 4.2. Sample Collection and Analysis

#### 4.2.1. Sample Collection

Food products taken for the cultivation of microbes were collected from different markets in Bangladesh, i.e., rural, peri-urban, and urban. The samples of food products were tomato, fish, and chicken. Food products were chosen based on expert opinions of the importance as a food source in the country, where fish and chicken (poultry) are common sources of protein from animals and tomatoes represent a common vegetable commonly consumed raw. All food samples taken were fresh and raw when sampled, see Table 7 for the specification of the amount of food sampled.

The number of samples to be taken from each outlet depended on the census. In markets with multiple vendors, the selection of samples was based on a systematic random method. An estimate of the total number of stalls was made and divided by the number of samples needed to get the proportion of stalls that should be sampled, i.e., if the total number of stalls were 40 and ten samples were required, every fourth stall was included. The stall nearest the market entry was to be the first stall sampled. Samples are considered unique if being from different vendors, except when a vendor sold more than one product of interest, where multiple samples from different food sources could be taken at the same stall.

#### 4.2.2. Microbial Analysis

In total, 249 fish, 366 tomato, and 359 chicken samples were collected. Samples were preserved in a cool box during transport. In the BLRI laboratory, fish samples were analysed for *V. cholerae* and *E. coli*, chicken samples for *Salmonella* spp., and tomatoes for *E. coli* and *Salmonella* spp.

Isolation and identification of *E. coli, Salmonella* spp. and *V. cholerae* were carried out through the conventional culture method, where fish were analysed for prevalence of *V. cholerae* and *E. coli*, chicken for *Salmonella* and tomato for *Salmonella* and *E. coli* using ISO 21872-1:2017, ISO-6579-1:2017, and ISO 16654:2001, respectively [56,57,58,59]. Briefly, to analyse *V. cholerae*, approximately 225 mL of alkaline peptone water (APW, Oxoid, UK) was added to 25 g fish sample and incubated for 18 ± 2 h at 37 °C ± 1 °C. In the following day, a loop-full of the incubated medium was streaked over thiosulfate citrate bile salts sucrose (TCBS, Oxoid, UK) agar and maintained for 24 ± 3 h at 37° ± 1 °C. After incubation, green color colonies on TCBS agar indicated *V. parahaemolytica,* while yellow color colonies indicated *V. cholerae*. Luria Bertani (LB, Oxoid, UK) agar was then used for streaking pure *V. cholerae* colonies. Oxidase testing was used for biochemical validation, and the pure isolates were stored in brain heart infusion broth (BHI, Oxoid, UK) with 15% glycerol at −20 °C for future use. *V. parahaemolyticus* strain ATCC 27969 and negative control strain *E. coli* strain ATCC 25922 were used as standard positive and negative controls.

For *Salmonella* identification, 25 g of food sample was homogenized with 225 mL sterile buffered peptone water (BPW, Oxoid, UK) using a stomacher at 200 rpm for ~45 s. The homogenized medium was incubated for 16–20 h at 37 °C as a pre-enrichment step. The following day, the pre-enriched sample (100 ul) was placed onto modified semi-solid Rappaport Vassiliadis (MSRV, Oxoid, UK) agar for 24–48 h at 41 ± 1 °C. After incubation, one loop full of MSRV was streaked on xylose lysine deoxycholate (XLD; Oxoid, UK) and another loop for MacConkey (Oxoid, UK) agar plates. These selective agar plates were incubated for 20–24 h at 37 ± 1 °C. One to two typical colonies from each cultured agar were selected to be sub-cultured on nutrient agar (NA, Oxoid, UK) and incubated for 20–24 h at 37 °C. Suspected colonies were confirmed by using different biochemical tests, e.g., in triple sugar iron agar (TSI, Oxoid, UK) and motility indole Urea (MIU, Oxoid, UK). Positive *Salmonella* strains were stored in 15% glycerol BHI at −20 °C to −80 °C. *Salmonella Enteritidis* strain ATCC13076 and negative control strain *E. coli* strain ATCC25922 were used as standard positive and negative controls.

For coliform and *E. coli* enumeration, Brilliance™ *E. coli*/Coliform Selective Medium (Oxoid, UK) was used following ISO 9308-1: 2014 [60]. The spreading method using decimal dilution series was applied to count bacteria. After drying the surface of the agar plates, 0.1 mL of each of the two consecutive selected dilutions was pipetted onto the plate, and then spread thoroughly over the surface with a sterile spreader. Incubated plates were kept at 37 °C for 24 h. On the next day, the numbers of pink (coliform) and purple (*E. coli*) colonies were counted accordingly, then calculated to the original concentration (colony forming units-CFU) per gram of food. A positive control strain used was *E. coli* strain ATCC25922. Total coliform count CFU/g was transformed to LogCFU/g for analysis.

#### 4.2.3. Antimicrobial Resistant Analysis

Samples with positive cultivation were tested for antibiotic susceptibility (AST) against eleven different antibiotics, including beta-lactam with clavulanic acid, penicillin, tetracyclines, aminoglycosides, chloramphenicol, cephalosporins, folate pathway inhibitors, macrolides, and quinolones. In short, the Kirby-Bauer disc diffusion method was performed in compliance with the Clinical and Laboratory Standards Institute’s requirements [61]. Two to three fresh colonies were randomly collected and suspended in 3 mL of normal saline, and the suspension’s turbidity was standardized to match the 0.5 McFarland standards. The Mueller Hinton agar plate was inoculated thoroughly on the agar surface with the bacterial inoculum, and within 15 min, the antimicrobial discs were placed onto the plate. Results were determined following an incubation period of 16–24 h at 35–37 °C. By using an automated zone inhibition reader (Scan 4000), the diameter of the zone of inhibition surrounding the discs was measured, and the results were compared to the CLSI breakpoints for interpretation. All bacteria strains were subjected to a test for antibiotic susceptibility, however, one tomato sample positive for *E. coli* was missed for the antibiotic susceptibility testing.

#### 4.2.4. Questionnaires

In addition to taking food samples, vendors from shops selected were also asked to participate in a face-to-face interview. The interviews included questions about the type of stall, knowledge about how to handle foodstuff, and hygiene routines (Questionnaire in Supplementary Material).

### 4.3. Qualitative Interview

During December 2020, complementary key informant interviews (KII) were held with key persons working with AMR in Bangladesh. A questionnaire and an invite to a discussion over zoom were sent out to 15 persons. Seven persons participated through interviews via zoom where the questionnaire (Questionnaire in Supplementary Material) was filled in subsequently, and eight persons did not respond to the invite because of unknown reasons. Interviews were held in English, with the possibility to clarify questions in Bangla when needed. All interviews were held with the same interviewer, all participants were assured to be anonymous in this thesis.

Key persons were chosen based on their work assignments and insight of the current situation regarding AMR in Bangladesh. Persons from different work categories such as government workers from both human health and animal health departments, other specialized departments connected to animal health and food security, pharmaceutical companies, universities, laboratories carrying out analysis for AMR, and epidemiologists were asked to participate. The seven persons that participated were connected to the import and sales of antibiotics, specialist knowledge of poultry and fish, connected to governmental work regarding human health and animal health, and laboratory work within the livestock sector.

Participants were presented with up to 21 questions, dependent on which work assignments they had. Answers from the interview were inserted into a template to get an overview of similarities and differences between the answers, trying to identify shared opinions about AMU and AMR.

### 4.4. Data Management and Analysis

The data was entered into Excel, cleaned, and imported into STATA 14.2 (STATACorp LLC, College Station, TX, USA). The logarithm of colony counts was used for analyses, and when colonies were too numerous to count (TNTC) or zero, the value was set as missing. Descriptive analysis was carried out for all the participants and categorical variables were presented as frequencies and percentages. Univariable analyses were conducted using the Chi2 test, Fishers exact test, and the t-test (where appropriate), as well as logistic regression using the logit command. A *p*-value ≤ 0.05 was considered statistically significant. For the qualitative data, themes were generated to identify a topic for the narrative. In the process, important statements, or quotes, with their references, were identified and extracted.

## 5. Conclusions

Some of the key things that were highlighted in the study were that commonly consumed foods like fish, chicken, and tomatoes were highly contaminated with the three bacteria investigated; *E. coli, V. cholerae,* and *Salmonella* spp. In addition to high levels of bacterial contamination, more than half of the isolates were multidrug resistant. This is a matter of concern indeed as it not only provides an evidence of the imprudent use of antibiotics in the animal as well as an agricultural sector but also, raises questions about food safety by suggesting that such widespread contamination could result from careless handling or the introduction of pathogens during processing. The prevalence of AMR among foodborne bacteria is growing, and this study further underlines the public health consequences associated with these microbial hazards. Some of the factors that exacerbate the situation are OTC antibiotic sales, a lack of antibiotic awareness, and inadequate surveillance, particularly in the veterinary industry. There is a dearth of health infrastructure, as is the case in any LMIC, as well as a paucity of data that paints an accurate picture of antimicrobial use.

To combat AMR and contamination, strict food safety rules must be implemented, combined with a robust surveillance system that tracks antimicrobial usage in the human, animal, and agricultural sectors. Adequate monitoring and evaluation are required to determine whether or not the already established laws are being followed. Together with this, efforts should be made to raise public knowledge about food safety, antibiotic use, and antimicrobial resistance. The emphasis should also be on the development of novel antibiotics and alternative therapies.

## Figures and Tables

**Table 1:** Responders in the study divided by gender, age, highest education completed, study site, market type, type of product sold, and cooling of product.

	Female (%)	Male (%)	Total (%)
Total number of respondents in each category	15 (100%)	838 (100%)	853 (100%)
Age of respondents			
<20	1 (6.67%)	30 (3.58%)	31 (3.63%)
20–35	9 (60.00%)	420 (50.12%)	429 (50.29%)
36–50	5 (33.33%)	357 (42.60%)	362 (42.44%)
>51	0	30 (3.58%)	30 (3.52%)
N/A	0	1 (0.12%)	1 (0.12%)
Highest education completed			
Illiterate	7 (46.67%)	192 (22.91%)	199 (22.51%)
Primary	1 (6.67%)	325 (38.78%)	326 (38.22%)
Grade 5–10	2 (13.33%)	216 (25.78%)	218 (25.56%)
High school	0	44 (5.25%)	44 (5.16%)
Graduation and above	5 (33.33%)	61 (7.28%)	66 (7.74%)
Study site			
Rural	2 (13.33%)	250 (29.83%)	252 (29.54%)
Peri-urban	5 (33.33%)	250 (29.83%)	255 (29.89%)
Urban	8 (53.33%)	338 (40.33%)	346 (40.56%)
Market type			
Supershop	6 (40%)	115 (13.72%)	121 (14.19%)
Traditional	9 (60%)	723 (86.28%)	732 (85.81%)
Type of product sold			
Chicken	2 (13.33%)	315 (37.59%)	317 (37.16%)
Fish	0	213 (25.42%)	213 (24.97%)
Tomato	13 (86.67%)	310 (36.99%)	323 (37.87%)
Is the product cooled			
No	14 (93.33%)	697 (83.17%)	711 (83.35%)
Yes, unspecified	0	5 (0.60%)	5 (0.59%)
Yes, in cool box	0	52 (6.21%)	52 (6.10%)
Yes, open to the environment but on ice	1 (6.67%)	84 (10.02%)	85 (9.96%)

**Table 2:** Microbial contamination in different food types and areas in Bangladesh.

Sample Type and Bacteria	No. of Positive/Total Samples Tested by Areas (%)			
	Rural	Peri-Urban	Urban	Overall
Tomato				
*Salmonella* spp.	15/96 (15.63%)	2/96 (2.08%)	10 /174 (5.75%)	27/366 (7.38%)
*Escherichia coli*	10/96 (10.42%)	29/96 (30.21%)	13/174 (7.47%)	52/366 (14.21%)
Chicken				
*Salmonella* spp.	25/95 (26.32%)	14/94 (14.89%)	23/170 (13.53%)	62/359 (17.27%)
Fish				
*Escherichia coli*	27/61 (44.26%)	22/65 (33.85%)	18/123 (14.63%)	67/249 (26.91%)
*Vibrio cholerae*	24/61 (39.34%)	26/65 (40.00%)	62/123 (50.41%)	112/249 (44.98%)
Overall	101/409 (24.69%)	93/416 (22.36%)	126/764 (16.49%)	320/1589 (20.14%)

**Table 3:** Cut-off values for disc diffusion test for antimicrobial susceptibility. Unit µg/mm (except for penicillin; units/mm).

Antibiotic	S	I	R
Amoxicillin + clavulanic acid	≥18	14–17	≤13
Cefixime	≥19	16–18	≤15
Ceftriaxone	≥23	20–22	≤19
Ceftriaxone	≥18	13–17	≤12
Erythromycin	≥23	14–22	≤13
Gentamicin	≥15	13–14	≤12
Streptomycin	≥15	12–14	≤11
Penicillin G	≥29	-	≤28
Tetracycline	≥15	12–14	≤11
Sulfamethoxazole + trimethoprim	≥16	11–15	≤10
Nalidixic acid	≥19	14–18	≤13

**Table 4:** The number of isolates from chicken, fish, and tomatoes from markets in Bangladesh that were resistant to different antibiotics.

	Tomato				Chicken		Fish					
	*Salmonella* spp.		*Escherichia coli*		*Salmonella* spp.		*Escherichia coli*		*Vibrio cholerae*		Total	
Total isolates	27		51		62		67		112		319	
Amoxicillin + Clavulanic acid	10	37.0%	11	21.6%	23	37.1%	10	14.9%	25	22.3%	79	24.8%
Cefixime	0	0.0%	6	11.8%	3	4.8%	14	20.9%	23	20.5%	46	14.4%
Ceftriaxone	0	0.0%	4	7.8%	4	6.5%	7	10.4%	24	21.4%	39	12.2%
Chloramphenicol	4	14.8%	15	29.4%	13	21.0%	26	38.8%	9	8.0%	67	21.0%
Erythromycin	17	63.0%	35	68.6%	54	87.1%	30	44.8%	36	32.1%	172	53.9%
Gentamicin	5	18.5%	4	7.8%	13	21.0%	12	17.9%	27	24.1%	61	19.1%
Streptomycin	7	25.9%	40	78.4%	33	53.2%	44	65.7%	39	34.8%	163	51.1%
Penicillin	20	74.1%	51	100.0%	60	96.8%	67	100.0%	112	100.0%	310	97.2%
Tetracycline	17	63.0%	35	68.6%	58	93.5%	46	68.7%	16	14.3%	172	53.9%
Sulfamethoxazole + Trimethoprim	8	29.6%	24	47.1%	44	71.0%	34	50.7%	24	21.4%	134	42.0%
Nalidixic acid	6	22.2%	14	27.5%	31	50.0%	24	35.8%	25	22.3%	100	31.3%
Multidrug resistance	16	59.3%	48	94.1%	59	95.2%	58	86.6%	50	44.6%	231	72.4%

**Table 5:** The number of samples classified as multidrug-resistant (MDR) isolated from food items in markets in Bangladesh.

	Positive Samples from Cultivation/Total Samples Analysed	Number MDR of Positive Samples (%)	Prevalence of MDR out of All Samples Analysed (%)
Tomato			
*Salmonella* spp.	27/366	16/27 (59.25%)	16/366 (4.37%)
*Escherichia coli*	51/366	48/51 (94.12%)	48/366 (13.11%)
Chicken			
*Salmonella* spp.	62/359	59/62 (95.16%)	59/359 (16.43%)
Fish			
*Escherichia coli*	67/249	58/67 (86.56%)	58/249 (23.29%)
*Vibrio cholerae*	112/249	50/112 (44.64%)	50/249 (20.08%)
Total	319/1589	231/319 (72.41%)	231/1589 (14.53%)

**Table 6:** Multiple-antibiotic resistance (MAR) index of the different bacteria in food samples from markets in Bangladesh.

	Isolates	MAR Index < 0.25	MAR Index 0.26–0.5	MAR Index 0.6–0.75	MAR Index > 0.75
Total	319	83 (26.0%)	144 (45.1%)	87 (27.3%)	5 (1.6%)
Tomato					
*Salmonella* spp.	27	11 (40.7%)	9 (33.3%)	7 (25.9%)	0
*Escherichia coli*	51	3 (5.8%)	33 (64.7%)	15 (29.4%)	0
Chicken					
*Salmonella* spp.	62	3 (4.8%)	31 (50%)	25 (40.3%)	3 (4.8%)
Fish					
*Escherichia coli*	67	7 (10.4%)	41 (61.2%)	19 (28.4%)	0
*Vibrio cholerae*	112	59 (52.7%)	30 (26.8%)	21 (18.7%)	2 (1.8%)

**Table 7:** The instructions for collecting food samples from traditional and modern markets in Bangladesh and the handling of samples in the lab before analyses.

Food	Fresh Fish	Chicken (Poultry)	Tomato
Approximate amount collected	~300 g edible portion per vendor	>300 g broiler per vendor	~150–300 g tomatoes per vendor
Sampling procedures	Several smaller fish (if <3–6 cm wide), one whole fish, or small pieces of multiple larger fish	One small whole chicken, or ½ of a larger chicken	2–4 tomatoes, depending on size
Preparing samples at laboratory	Fish meat was collected by cutting at 3 to 4 different sites of the fish collected (approximate total of 50 g); excluding all bone, gills, intestine, and fluid. Fish meat was then cut into small pieces and homogenized before weighing 25 g as sample.	Chicken carcass (whole or a part) was collected by cutting from 3 to 4 different sites of the body (approximate total of 50 g); excluding bone. Chicken meat was then cut into small pieces and homogenized before weighing of 25 g as sample.	Tomato samples were prepared by cutting the tomatoes into small pieces (approximate total of 40–50 g), then and homogenized before weighing of 25 g as sample.

Note: Fish were of pangash type and from aquaculture, but the vendors were not asked to verify this upon sampling. Chicken came from different types of housing, but vendors were not asked to specify this during sampling.

## Data Availability

Data will be available upon request.

## Supplementary Materials

The following supporting information can be downloaded at: https://www.mdpi.com/article/10.3390/antibiotics12030555/s1, Supplementary Material S1: Questionnaire for vendors. Supplementary Material S2: Interview guide for key informants.
