# Supplementary material for: Microbial Contamination and Antibiotic Resistance in Marketed Food in Bangladesh: Current Situation and Possible Improvements
Source: Antibiotics (Basel). 2023 Mar 10;12(3):555. doi: 10.3390/antibiotics12030555 (PMC10044357; doi:10.3390/antibiotics12030555)
Supplement: Supplementary file 1 [file antibiotics-12-00555-s001.zip › Supplement S1 questionnaire for vendors.pdf]

Questionnaire number:

Vendor Questionnaire – Bangladesh Food Safety

☐ **Consent provided to participate in study (*attach signed consent form*).**

1. Name and contact information for the respondent (vendor)

1.1	Name of interviewer			1.2	Date	
1.3	Town	1.4	Thana	1.5	Upazila	
1.6	District			1.7	Market	
1.8	GPS North			1.9	East	
1.10	Name of the respondent			1.11	Phone *(optional)*	

2. Basic demography

2.1	Gender of respondent (1=male, 2=female)		2.2	Age (1<20, 2=20-35, 3=36-50, 4>51 years)	
2.3	Type of product(s) sold by respondent (1=fresh fish, 2=tomato, 3=broiler 4=other)	*(if other, please specify)*			
2.4	Does this vendor sell any ready-to-eat products? (1=Yes, 0=No)				
2.5	***(If yes)*** Please describe which:				
2.6	Years of selling current products in markets				
2.7	Highest education completed (0=none, 1=primary, 2=class 5-10, 3=Higher secondary (11-12), 4=Graduation and above, 99=Not asked or not able to assess)				
2.8	Are you member of a traders association (1=Yes, 0=No)?				
2.8a	**(*If yes*)** Give the name of the traders association(s):				

3. Sales

	Complete all that apply for this vendor:	a. Broiler	b. Tomato	c. Pangash<br>frozen solid<br>fresh/on ice<br>(*circle 1*)
3.1	Approximately how much of these products do you sell in one day (on average)?	kg/day	kg/day	kg/day
3.2	In general, from whom do you procure the raw products (1=own production, 2=producer, 3=wholesale market, 4=middleman, 5=other________________)			
3.3	What time in the morning do you usually collect, harvest, or procure the products?	am/pm	am/pm	am/pm
3.4	What time do you usually open the stall?	am/pm	am/pm	am/pm

Questionnaire number:

3.5	What time do you close the stall for the day?	am/pm	am/pm	am/pm
3.6	What do you do with the remaining products that you cannot sell? (1=sell to a third party, 2= preserve for the next day, 3= consume, 4=other_____________________)			
3.6a	***(If 1)*** To whom do you sell the products? (1=mobile vendor(s), 2=restaurant, 3=trader, 4=other______________________)			

4. Vendor knowledge and attitude towards food safety

4.1	Do you think it is possible to get sick from eating this kind of product, in general? (1=Yes, 0=No, 99=don't know)	
4.1a	(***If yes***) Please mention the diseases you can get:	
4.2	Do you worry more about chemicals or germs in food? (1=chemicals, 2=germs, 99=don't know)	
	Do you agree with the following:	agree=1,/ disagree=0/ Don't know=99
4.3	The longer a raw product has been at the market, the lower the quality.	
4.4	It does not matter how old the food is if you cook it well.	
4.5	I can tell by the look and smell of food if it is safe to eat.	
4.6	I sometimes eat raw food (such as vegetables) without cooking it first.	

5. Purchase the product, then make the following *vendor observations*.

5.1	Is tomato/fish/poultry meat in this stall on ground or elevated? (2=elevated on table, 1=elevated in baskets or bins, 0=directly on ground)	
5.2	Is tomato/fish/poultry meat uncovered? (1=Yes, 0=No)	
5.3	Is there any cooling? (3=refrigerator, 2=cool box, 1=open to environment, but on ice, 0=No)	
5.4	Are there flies on the food products in this stall? (3=innumerable, 2>10, 1 ≤10, 0=No)	
5.5	Do the tomatoes/fish/poultry meat look clean? (1=Yes, 0=No, there is visible dirt)	
5.6	Do utensils look clean? (1=Yes, 0=No) *Circle all that apply: knife, blade, cutting board, scale.*	
5.7	*(Produce only)* Are products rinsed, sprayed or wiped during sale? (1=Yes, 0=No)	

Questionnaire number:

Household Consumer Questionnaire – Bangladesh Food Safety

☐ **Consent provided to participate in study (*attach signed consent form*).**

1.1	Name of interviewer				1.2	Date	
1.3	Town		1.4	Thana	1.5	Upazila	
1.6	District				1.7	Market	
1.8	GPS North				1.9	East	

7.1	Type of product(s) purchased by respondent (1=fresh fish, 2=tomato, 3=poultry, 4=other)	*(if other, please specify)*

8. Name and contact information for the customer responding

8.1	Name of the customer		8.2	Phone *(optional)*	
8.3	Gender of customer (1=male, 2=female)		8.4	Age (1<20, 2=20-35, 3=36-50, 4>51 years)	
8.5	Highest education completed (0=none, 1=primary, 2=class 5-10, 3=higher secondary (11-12), 4=graduation and above, 99=Not asked or not able to assess)				

9. Consumption of *circle one: / /* product(s)

		**fish**	**tomato**	**poultry**
9.1	What is the total number of people in your household?	people	people	people
9.2	Are there people in your household who do not consume this product?	people	people	people
9.3	In grams, how much was consumed in total yesterday (or most recently)?	g	g	g
9.4	How often do you consume this product?	/week *or* /month	/week *or* /month	/week *or* /month
9.5	Have you ever eaten this product raw or undercooked? (1=Yes, 0=No)			
9.6	Which market/retail type do you often go to buy this product? (1= **traditional** uncovered, open air, roadside or rickshaw, 2=**traditional** municipal market, 3=**modern** supermarket/convenience store)			

10. Attitudes towards food safety and the market

	Do you agree with the following:	agree=1,/ disagree=0/ Don’t know=99

Questionnaire number:

10.1	The longer uncooked food has been at the market, the lower the quality.	
10.2	Improper handling of food by the seller could make people sick.	
10.3	Germs are on the skin, nose and mouth of everyone.	
10.4	The cleanliness of sellers is important when I choose where to buy food.	
10.5	Diarrhea can be transmitted through food.	
10.6	I worry more about chemicals in the food than germs.	

11. Food safety practices *(use "99" if respondent is not familiar with the cooking)*

11.1	For the most recent consumption, how long was the tomato/fish/poultry meat kept in your house before preparing? (*enumerator to indicate whether hours or days)*	
11.1a	***If >1 hour,*** Where/how do you store the uncooked tomato/fish/poultry meat at home? (1=Refrigerator, 2=ambient temperature, 3= Other)	
11.2	Did you wash the product before preparation? (1=Yes, 0=No)	
11.3	Did you chop or cut the product before preparation? (1=Yes, 0=No)	
11.4	Did you cook the product? (1=Yes, 0=No)	
11.4a	(***If yes)*** How long (minutes)?	Min
11.5	Which dishes do you often prepare from this product? (***specify all that apply***: 1=salad, 2=roast, 3=fry, 4=grill, 5=boiled/soup, 6=stew/curry, 7=other)	
11.6	In general, do you use the same **knives** for both cooked and raw products, such as salad vegetables? (1=Yes, 0=No)	
11.7	Do you wash knives between use? (1=Yes, 0=No)	
11.8	Do you use the same **cutting boards** for both cooked and raw products, such as salad vegetables? (1=Yes, 0=No)	
11.9	Do you wash cutting boards between use? (1=Yes, 0=No)	
11.10	Do you wash your hands *with soap* before handling food. (1=Yes, 0=No)	
11.11	Do you wash your hands *with soap* after handling raw meat/fish. (1=Yes, 0=No)	

12. Food safety and the home: storage and leftovers

	Do you agree with the following:	agree=1,/ disagree=0/ Don't know=99
12.1	Improper food storage is dangerous to health.	
12.2	Raw fish and meat should be stored in a cold place.	
12.3	Germs can get into food from insects and pests.	
12.4	I can tell by the look and smell of food if it is safe to eat.	
12.5	High temperature or freezing is a safe method to destroy germs.	

12.6	I sometimes worry about eating leftovers because they can make me sick.	
12.7	I always cook leftovers again before eating them.	
