# Supplementary material for: Microbial Contamination and Antibiotic Resistance in Marketed Food in Bangladesh: Current Situation and Possible Improvements
Source: Antibiotics (Basel). 2023 Mar 10;12(3):555. doi: 10.3390/antibiotics12030555 (PMC10044357; doi:10.3390/antibiotics12030555)
Supplement: Supplementary file 1 [file antibiotics-12-00555-s001.zip › Supplement S2 Questions for key informant interviews.pdf]

Questions to stakeholders regarding antimicrobial use and antimicrobial resistance in Bangladesh

Begin with background information:

Gender: Filled by interviewer

Dependent of current occupation, different forms?? Most important to labs/stakeholders involved in carrying out tests for AMR?

1. What is your occupation?
2. What is your work responsibility?
3. How long have you worked in this role?

For practitioners/pharmaceuticals

4. Do you prescribe/recommend antibiotic use in your profession, or sell antibiotics directly to the user?
5. Do you ever prescribe or sell antibiotics without seeing the sick animal? Do you ever recommend or sell antibiotics to animals that are not sick?

6. To which types of animal do you most often prescribe antibiotics?

7. How do the drugs reach the farmer? (Alternatives; leaving them there, farmer buy at pharmacy etc)

	Very common	Not so common
Leave them at the farm		
Farmer buy at pharmacy		
Farmers get them from reseller		
Other:		
Other:		

8. Which is the most common disease to treat with antibiotics? (→ open answer or alternatives like diarrhea, respiratory disease, mastitis etc)

List diseases	Which antibiotics do you treat them with commonly?	Do you ever need to change to another medicine for this?

a. Which antibiotic do you treat it with? (open answer or alternative?)

b. Do you change antibiotic sometimes? Why? (open answer or alternative)

9. Do you know if there any national guidelines for ab use?

   a. If yes, do you know what they say?

   b. If yes, do you think people know about them?

   c. If no, do you think there should be guidelines, and what should there be in them?

10. Do you often se failure of treatment when antibiotics are used?

11. Do you think antimicrobial resistance (AMR) is a problem in Bangladesh?

    a. If yes, is it possible to change the situation?

    b. What needs to be done to improve the situation? (Multiple choice; education, better guidelines, surveillance etc)

Improvements	Very important	Minor important
Education of farmers		
Education of stakeholders		
Better guidelines		
Surveillance		
Other:		
Other:		

    c. Do you have any responsibility for monitoring the AMR-situation in your profession?

12. Do you know if surveillance and testing regarding AMR are carried out in Bangladesh?
    a. If yes; is it enough/does it have to be further developed
    b. If no; would it be good?

For stakeholders carrying out testing for AMR:

13. Which kinds of antibiotics are tested (multiple choice of different antibiotics)
14. What are the organisms you tested to identify the AMR trend?
15. How is the situation based on available tests done?
    a. Do you see resistance patterns?
    b. Do you see signs of multidrug resistance?
16. Are findings shared with other stakeholders?

17. Is testing done at regular basis?
18. Would it be possible to do more testing? (In terms om enough capacity at labs etc)
19. Which are the biggest challenges in implementing a/improving a surveillance programme? (Multiple choice; capacity for analysis, money, hard to get samples)

Please also give your opinion on these two overarching questions:

1. *What are the barriers in implementing better antibiotic use?*

2. *What are the barriers in implementing increased surveillance?*

